# Effects of 6-Week Supplementation with GliSODin on Parameters of Muscle Damages, Metabolic, and Work Performance at International Level Rowers after Specific Maximal Effort

**DOI:** 10.3390/biology11101437

**Published:** 2022-09-30

**Authors:** Olina Dudašova Petrovičova, Ivan Stanković, Neda Milinković, Violeta Dopsaj, Brižita Đorđević, Milivoj Dopsaj

**Affiliations:** 1 Faculty of Pharmacy, University of Belgrade, Vojvode Stepe 450, 11221 Belgrade, Serbia; 2 Faculty of Sport and Physical Education, University of Belgrade, Blagoja Parovića 156, 11000 Belgrade, Serbia; 3 Institute of Sport, Tourism and Service, South Ural State University, 60 Sony Krivoy Str., 454080 Chelyabinsk, Russia

**Keywords:** athletes, oxidative stress, superoxide dismutase, GliSODin, exhaustive exercise

## Abstract

**Simple Summary:**

Intensive physical activity can cause some deleterious effects on athletes’ health and sports performance. One of the main reasons for these effects seems to be oxidative stress. Therefore, this study was conducted to see if supplementation with an enzymatic antioxidant containing superoxide dismutase of plant origin, GliSODin, could reduce some negative effects of oxidative stress connected to exhaustive exercise. According to the results of this study, it was concluded that supplementation with GliSODin can protect athletes from muscle damage and decrease inflammation caused by intensive physical activity and have some positive influence on the sports performance of elite rowers. Therefore, more studies with a larger number of participants are needed to confirm this positive effect of GliSODin supplementation.

**Abstract:**

This study aimed to investigate the effect of supplementation with plant origin superoxide dismutase (SOD), GliSODin, on parameters of muscle damage, metabolic, and work performance at international level rowers. Twenty-eight rowers were included in a randomized, double-blind study. The study was conducted during a 6-week preparation period. At the beginning of the study and after 6 weeks of the supplementation period, all rowers were tested on a rowing ergometer. Blood samples were taken from the antecubital vein before and after every ergometer testing. Muscle damage markers creatine kinase (CK) and lactate dehydrogenase (LDH), total antioxidant capacity (TAC), inflammation parameters interleukin-6 (IL-6), and C-reactive protein (CRP) were measured. Rowing performance was assessed by lactate level in capillary blood and power output on the rowing ergometer. After supplementation, experimental group had significantly lower CK (*p* = 0.049) and IL-6 (*p* = 0.035) before and IL-6 (*p* = 0.050) after exhausting exercise on ergometer. Relative change of power output at 4 mmol/L concentration of lactate in blood, considering the initial and final test, was significantly higher (*p* = 0.020) in the supplemented group. It was concluded that GliSODin could be considered a good supplement in preventing some deleterious effects of intensive physical activity, including inflammation and muscle damage, and consequently, to enable a better rowing performance of elite rowers.

## 1. Introduction

Clinical, epidemiological, and basic research evidence clearly suggests that the implementation of regular physical activity can enhance overall health. It has preventative and therapeutic effects on many chronic disorders like cardiovascular, metabolic, pulmonary, and immunity disorders [1,2]. On the other hand, it has been proven that a high load of physical activity can cause some serious side effects such as muscle damage, hormone disbalance, lower immunity, and the occurrence of oxidative stress. Oxidative stress is considered the main cause of many negative effects of intensive exercise [3]. Oxidative stress is defined as the “disturbance of the oxidation-reduction balance in favor of oxidants, leading to a disturbance in redox signaling and control and molecular damage” [4]. Reactive oxygen species (ROS) are very reactive and the most common type of free radicals synthetized in our body besides reactive nitrogen (RNS) and sulfurous species (RSS) [5]. ROS are synthetized in small quantities during normal metabolic reactions and they have many important regulatory roles in an organism, including the immune system, antioxidant defense system, and cell signaling.However, excess amounts of free radicals can easily react with essential molecules such as proteins, lipids, carbohydrates, and DNA causing damage or even death of a cell [6]. As ROS synthesis is part of usual oxygen metabolism, our organism has developed an antioxidant defense system to control the amounts of free radicals. This defense system is conducted of antioxidant enzymes such as superoxide dismutase (SOD), glutathione peroxidase (GPX), glutathione reductase (GR), catalase (CAT), and nonenzymatic antioxidants including vitamin C, vitamin E, glutathione, β carotenes, vitamin A. When the production of free radicals overwhelms the capacity of our antioxidant defense system, oxidative stress occurs [5,7].

Physical activity can cause oxidative stress in a dosage-dependent manner; more intensive exercise provokes more stress. The main source of ROS during physical activity is elevated electron leakage from respiratory chain complexes in mitochondria due to increased oxygen consumption. In addition, ischemic reperfusion of the active muscle and NADPH-oxidase-producing free radicals in phagocytes is another source of ROS [7]. It is known that regular physical training up-regulates antioxidant defense systems and elite athletes reduce exercise-induced oxidative stress more effectively [8,9,10]. Despite this adaptation mechanism in the periods of intensive training during preparation and competing season elite athletes can develop oxidative stress. Oxidative stress is most probably connected with overtraining syndrome leading to higher injury accidence and decreased sports performance [11].

Over the last few decades, an important focus of sports and nutrition scientists has been antioxidant supplementation for athletes to overcome the consequences of exercise-induced oxidative stress. Still, there is no unique answer to this problem. Some studies confirmed positive effects, especially when multiple antioxidant sources are combined, suggesting a synergistic effect of vitamins and bioflavonoids [12,13]. Other studies did not find any effect of supplementation, mostly concerning vitamin C and E supplementation [14,15]. However, some scientist has noticed the negative influence of antioxidant supplementation in particular due to decreased exercise-induced adaptation [16,17]. Antioxidants used in these studies were mainly nonenzymatic molecules such as vitamin C, vitamin E, vitamin A, ALA, and some phytochemicals like quercetin and concentrated vegetable and fruit juice. Any further information can be found in the listed references [12,13,14,15,16,17]. A very interesting approach to this problem is the usage of antioxidants that could enhance an organism’s own endogenous antioxidant capacity, like enzymatic antioxidants. Supplements containing SOD can trigger the endogenous antioxidant machinery by stimulating other antioxidant enzymes, including CAT and GPx, by enhancing oxidative stress signals. SOD catalyzes the reduction of highly reactive superoxide anion in less reactive hydrogen peroxide. Hydrogen peroxide is further neutralized into the molecules of oxygen and water by the activity of CAT and GPX. This seems to be an advantage over nonenzymatic antioxidants in neutralizing free radicals in a more effective way.

This study aimed to evaluate the effects of supplementations with enzymatic antioxidants based on plant-origin superoxide dismutase, GliSODin. We hypothesized that supplementation with GliSODin would improve the endogenous antioxidant defense system, limit oxidative stress leading to decreased muscle injury and inflammatory response and enhance performance during exercise in elite rowers as a typical power endurance load sport.

## 2. Materials and Methods

This double-blinded, placebo-controlled human study was approved by the Ethical committee of the Faculty of Pharmacy, Belgrade University (protocol No. 2192/2). At the beginning of the study, all participants were informed orally and in written form about the nature of the investigation and gave their written consent to participate in the study.

### 2.1. Subjects

Twenty-eight international category rowers participate in this study. Basic anthropometric and training data are shown in Table 1. The sample size was predetermined using G-power software version 3.1.9.4. According to the assumed change of measured parameters based on some similar studies with the same supplementation in a similar group of athletes, we calculated that 28 participants are enough to conduct this kind of study with the actual power of 0.96, which was high enough to run this study. Including criteria: healthy athletes without any chronic diseases such as diabetes, cardiovascular, renal, or gastrointestinal diseases or surgical procedures in the last 6 months that could interfere with a training regime, both genders, 18 to 30 years old. Exclusion criteria: allergy to ingredients of the tested supplement, gluten intolerance, supplementation with other supplements two weeks before the study. All participants were on a regular diet and had not been taking any other supplements during the study. The athletes were asked to inform scientific staff if they had been taking some other nutritional supplements or medicines during the study.

### 2.2. Experimental Procedure

Participants in this study were randomly divided into experimental (*n* = 15) and control group (*n* = 13). The experimental group was supplemented with GliSODin and the control group received the placebo. Supplementation implied consummation of 500 mg of GliSODin, two capsules each containing 250 mg, once daily during the 6-week period. The experimental group took their supplement one hour before training or one hour before breakfast on days without training. The dose of supplementation was determent according to the manufacturer’s recommendation. The pharmacokinetic profile of this supplement is not yet done; the manufacturer recommended supplement to be taken on an empty stomach before breakfast. We considered that one hour before training is optimal timing because it would enable neutralization of free radicals produced during training and athletes habitually do not eat in this period before training and they were additionally asked to comply with this recommendation. GliSODin is an original antioxidant formula made of special melon (Cucumis melo LC, *Cucurbitaceae*) extract rich in SOD combined with biodegradable protein gliadin, isolated from wheat, manufactured by ISOCELL NUTRA S.A.S (Paris, France). The composition of the supplement is displayed in Table 1. This combination is gastroresistant, so it has enabled efficient delivery and resorption of enzymes in the small intestine by the oral route [18]. The control group took two capsules in the same period of the day; the capsules had the same appearance but contained only maltodextrin. Supplementation was conducted during 6 weeks of mesocycle of basic preparation with habitual physical training work.

On the first day of the study, before supplementation, and at the end of the 6-week supplementation period exercise performance of all participants was tested on a rowing ergometer. Before every testing on the ergometer, all rowers had 24 h resting period as part of recuperation. A test on an ergometer was conducted with increased interval exercise load. For males, it was 2 min rowing session with increasing load for every session with the following protocol: 150 W (watt), 200 W, 250 W, 300 W, 400 W, and individual maximal effort, with 5 min pauses between the first two session attempts, 6 min between second two session attempts, and 8 min pause before maximal effort attempt. For females, the load increased with the following protocol: 150 W, 180 W, 220 W, 250 W, 300 W, and individual maximal effort with the same pause schema. The rowers’ trainers and medical technicians, besides the research team, were monitoring the test. The same test was conducted after 6 weeks of supplementation.

During these 6 weeks, the rowers were training according to the schedule determined by their trainers, once daily, 6 days a week. The duration of every training session during the study was 1 h 52 min ± 12 min. Moreover, they were advised to adhere to the common diet without any additional supplementation. To define the selected blood parameters change during the specific testing load, vein blood samples were taken at initial and final (after 6 weeks of supplementation) ergometer testing. Blood samples were taken 20 min before warm-up for ergometer testing when rowers were at a resting state. The second blood sampling was 10 min after a specific testing load to examine changes in blood parameters after intensive physical activity. During the ergometer testing, the rowers were allowed only to drink water.

Blood samples were taken from the antecubital vein using a BD Vacutainer^®^ tube without additives for serum separation.

### 2.3. Measurements

Collected blood samples were centrifuged at 3000 RPM for 10 min to separate serum; serum aliquots were stored at −20 °C until analyzed.

Total antioxidant capacity (TAC) was determined using an automated method developed by Erel [19]. This method is based on the de-coloration of 2.2′-azinobis-(3-ethylbenzothiazoline-6-sulphonic acid radical cation (ABTS) by antioxidants present in serum. The intensity of color change was measured using the Olympus AU-400 Biochemical Analyzer (Beckman Coulter Inc., Brea, CA, USA). The Trolox (a water-soluble analog of vitamin E, 6-hydroxy-2.5.7.8-tetramethylchroman-2-carboxylic acid) was used for calibration of reaction rate. Sample TAC values were expressed as mmol Trolox equivalent/L.

Creatine kinase (CK) and lactate dehydrogenase (LDH) activity in serum were measured using the spectrophotometric method with commercial reagents on Olympus AU-400 Biochemical Analyzer (Beckman Coulter Inc., Brea, CA, USA) expressed in units per liter (U/L).

Serum samples were used to determine C- reactive protein (CRP) concentration on Olympus AU-400 Biochemical Analyzer (Beckman Coulter Inc., Brea, CA, USA) by an immunoturbidimetric method using commercial reagents, expressed in mg/L.

Interleukin-6 (IL-6) concentration in serum was measured using a commercially available ELISA kit (R&D Systems, Inc., Minneapolis, MN, USA) following the manufacturer’s instructions. Concentration was determined using a spectrophotometric Microplate reader (T-6100, Life and Analytical Sciences, Rayto, China) on 450 nm. We have used a highly sensitive kit (the limit of detection is 0.2 pg/mL), and the concentration of IL-6 is expressed in pg/mL.

Lactate concentration was measured for every rower during ergometer testing in capillary blood samples from a finger prick using a hand-handled automatic testing device. The samples were taken as follows during ergometer testing: before testing in rest (as an initial lactate level) and 1 min after each 2-min rowing session. We have been using Lactate Plus hand-held testing devices and matching test strips made by Nova Biomedical. Lactate concentration was expressed in mmol/L. La peak was a peak concentration of lactate in capillary blood after maximal effort attempt measured from samples taken in 1, 3, or 5 min in rest. The power of rowing at 4 mmol/L of lactate (W at 4 mmol/L La) was calculated as the relation of achieved watts and the lactate by stages of the incremental test protocol using a regression square curve according to previously published research [20,21]. W max was an achieved mean value of power output in Maximal effort at the last test stage. Participant rowing performance was tested on a wind-resistance-braked rowing ergometer (Model D; Concept2, Morrisville, VT, USA). Rowers were familiarized with laboratory exercise testing procedures before the administration of tests. Before initial and final testing (after 6 weeks of supplementation), all rowers had a self-paced warm-up lasting 5 min on the same ergometer, 10 min before testing. Graded exercise power and maximal effort power of every participant were measured on ergometer machines expressed in Watt. Ergometer machines have monitors where we can continuously follow power output.

### 2.4. Statistical Analyses

All analyses were conducted using IBM SPSS v23.0 statistical software. As a main statistical procedure for differences between groups and tests, we used the MANOVA and ANOVA repeated measure 2 × 2 protocol as a suitable design for our study. The influence of supplementation on the difference in and between the groups, Partial Eta^2^ was calculated. Difference of average values of variables in relation to the tests in every group was expressed like delta values in % (ΔCK, ΔLDH, ΔTAC, ΔIL-6, ΔCRP). Delta value is calculated according to the formula: ((Test2/Test1) − 1)·100, where Test1 is the average value of the variable before ergometer testing for a particular group, and Test2 is the value of the variable after ergometer testing for the particular group during initial and final testing. Difference of average values of variables of rowing performance considering groups (experimental and control) was expressed like delta values in % (ΔLa peak, ΔW max, ΔW at 4 mmol/L La). Delta value is calculated according to the formula: ((Test2/Test1) − 1)·100, where Test1- is the average value of the variable before supplementation on ergometer testing for a particular group and Test2—the value of the variable after supplementation on ergometer testing. The level of statistical significance between variables was defined based on criterion *p* ≤ 0.05 [22].

## 3. Results

### 3.1. Anthropometric and Training Data of Study Participants

Both groups of rowers, experimental and control, were uniform according to the anthropometric characteristics, including age, mean height, mean weight, as well as years of training, and training session duration, as shown in Table 2.

### 3.2. Biochemical Parameters

In Table 3, we can see changes in measured biochemical parameters as the result of intensive physical activity during the initial test on the rowing ergometer. General differences between groups were not observed (Wilks’ Lambda Value = 0.855, *p* = 0.599, Partial Eta^2^ = 0.145). Considering CK and LDH as parameters of muscle damage we can see there was no differences between initial values of CK and LDH in groups, (CK *p* = 0.418, Part. Eta^2^ = 0.025; LDH *p* = 0.891, Part. Eta^2^ = 0.001). Physical activity induced rise of both parameters in similar way in groups (CK *p* = 0.426, Part. Eta^2^ = 0.025; LDH *p* = 0.662, Part. Eta^2^ = 0.007). Total antioxidant capacity was slightly decreased 10 min after physical activity without any statistically significant differences according to the groups before and after physical activity (before test *p* = 0.370, Part. Eta^2^ = 0.031; after test *p* = 0.974 Part. Eta^2^ ≤ 0.001). Parameters of inflammation CRP and IL-6 were elevated after exercise.

Table 4 shows the final results of measured values after 6 weeks of supplementation. General multivariate linear model shows there is some significant differences between measured parameters after supplementation according to groups before exercise (Wilks’ Lambda Value = 0.515, F = 4.151, *p* = 0.008, Partial Eta^2^ = 0.480). The initial value of CK was significantly lower in the experimental group (*p* = 0.049) same as the initial values of IL-6 (*p* = 0.035) after 6 weeks of supplementation compared to the control group. Other measured parameters, including LDH, TAC, and CRP, did not differ significantly. Established value of Partial Eta^2^ = 0.062 for LDH indicates lower resting values in the experimental group in the final test. After ergometer testing, all values followed the trend of change same as during testing before supplementation, and there were no generally significant differences between values according to groups (WLV = 0.661, F = 2.253, *p* = 0.085, Partial Eta^2^ = 0.339). Specific maximal effort training has had the same influence on measured parameters according to groups (CK *p* = 0.139, LDH *p* = 0.245, TAC *p* = 0.162, CRP *p* = 0.897) except significantly lower values of IL-6 after testing in experimental group (*p* = 0.050). Partial Eta^2^ = 0.082 for CK shows a tendency for lower CK serum concentration in the experimental group at the final test after exercise, the same as Partial Eta^2^ = 0.074 for TAC indicates higher values in the experimental group.

Effects of maximal effort controlled physical activity in the form of ergometer testing of rowers on changes of measured parameters inside the groups are shown in Table 5. Results are presented as delta values expressed in the percentage of change of some parameters according to tests (Test1- before exercise, Test2- after exercise) during initial and final testing. There were no statistically significant differences between changes of parameters of muscle damage (ΔCK *p* = 0.919, ΔLDH *p* = 0.509), antioxidant capacity (ΔTAC *p* = 0.579), and observed inflammation parameters (ΔIL-6 *p* = 0.596, ΔCRP *p* = 0.763) between groups on initial testing before supplementation. Observing changes in parameters inside the groups influenced by ergometer testing after 6 weeks of supplementation, we can see there was a significant difference between changes in CK in the experimental and control group (*p* = 0.032). Changes in other parameters were without significant differences according to the groups. The third part of Table 4 shows delta differences (in %) between tests (initial and final) according to variables considering groups (experimental and control); this demonstrates the overall effect of supplementation on selected biochemical parameters in a group of elite rowers. Parameters of muscle damage and inflammation have reached statistically significant change (ΔCK *p* = 0.009, ΔIL-6 *p* = 0.031) considering groups through initial and final testing, while LDH, TAC, and CRP changes were without statistical significance.

Work physical performance of elite rowers was estimated using changes in La peak, W max, and W at 4 mmol/L La. All parameters of sports performance were similar in both groups before (initial test) and after supplementation (final test), as it is showed in Table 6.

**Figure 1:**
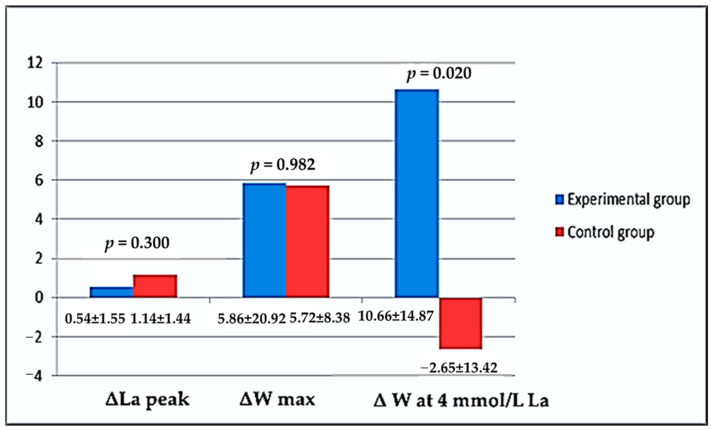
Descriptive results considering explored variables of sports performance according to the tests and supplementation groups expressed as a delta value (in % of differences).

In Figure 1, all delta differences (in %) between tests (Initial Test and Final Test) according to variables of rowing performance considering groups (experimental and control) are graphically shown. There were no statistically significant differences in La peak and W max values. On the other hand, there was a big change in W at 4 mmol/L La. In an experimental group, there was an increase in power output at 4 mmol/L concentration of lactate in capillary blood, unlike in the control group, where this value was significantly lower (*p* = 0.020).

## 4. Discussion

This study was undertaken to examine the effects of a new orally effective antioxidant supplement consisting of vegetable origin SOD chemically combined with wheat gliadin, GliSODin, on parameters of muscle damage, inflammation, and work performance in elite rowers during intensive physical activity. Twenty-eight elite international class rowers participated in the study, while supplementation was conducted during 6 weeks of mesocycle of the basic preparation period.

It is a fact that strenuous physical activity leads to oxidative stress. In 1978, Dillard et al. [23] were the first to demonstrate that physical exercise can lead to an increase in lipid peroxidation, which is one of the markers of oxidative stress. Oxidative stress is considered one of the most important factors that contribute to the development of muscle fatigue and damage during exercise [24,25]. The increased activities of CK and LDH enzymes in plasma are considered a biochemical marker of muscle damage and are commonly used in clinical practice [26,27]. Creatine kinase and LDH are also indicators of the degree of metabolic adaptation to the physical training of skeletal muscles. Both enzymes are part of muscle metabolism, and their serum concentration is normally very low; they increase notably after intensive exercise and in muscle pathology [28,29]. High serum CK activity is a consequence of damage to the sarcolemmal membrane [30]. The damage is probably proportional to the duration and intensity of the contraction and is related to the severity of muscle soreness [31]. In our research, CK serum concentration was higher after physical activity as we expected at the beginning of the study (173 ± 125 vs. 225 ± 152 in the experimental group, 209 ± 101 vs. 266 ± 109 in the control group) without statistically significant differences between groups. After 6 weeks of supplementation, the initial value of CK in the experimental group (152 ± 62 U/L) was significantly lower compared to the control group (215 ± 101 U/L); *p* = 0.049, Partial Eta^2^ = 0.137. After performance testing, the CK value was not significantly different, but Partial Eta^2^ = 0.082 pointed to the trend of lower values in the experimental group. This finding is in line with the previously published study indicating that the antioxidant effect of GliSODin had a protective influence on muscles. The same changes in resting level of CK were found in the study with college soccer players who, supplemented with the combined antioxidant supplement Resurgex Plus, containing 500 mg of GliSODin [32]. It was suggested that lower initial values of CK indicate that the supplement does not attenuate acute muscle breakdown in response to exercise but rather improves the rate of recovery. A study with Polish national class rowers, supplemented with GliSODin, also considered changes in CK and LDH after 6 weeks of supplementation. In this study, they noticed the same pattern of the rise of CK and LDH after maximal effort 2000-m time trial and lower initial value of CK after supplementation, but without significant differences between the groups. Plasma LDH activities have been reported to increase immediately or within 8 h after an exercise [33]. Lactate dehydrogenase serum concentration was higher after exercise in both groups (exp.gr. 148 ± 21 U/L vs.177 ± 22, cont.gr. 147 ± 16 U/L vs. 180 ± 17 U/L) in initial and final testing without any significant differences considering the groups. During the final test, Partial Eta^2^ = 0.0620 pointed to potentially significantly lower initial values of LDH in the experimental group. Including more biochemical parameters of muscle damage like myoglobin, aspartate aminotransferase (AST), alanine aminotransferase (ALT), and muscle biopsy could give a better insight into the GliSODin effects on muscle damage. Although these measurements overcome the limitation of this study, they are worthy of consideration for any further research work.

Total antioxidant capacity is part of the nonenzymatic defense system protecting our body against the excess amount of free radicals. Uric acid, the final product of purine metabolism, is thought to be the main part of this antioxidant system and the most important nonenzymatic plasma antioxidant [34]. According to Wayner, Burton, Ingold, Barclay, and Locke [35], uric acid determines about 35–65% of TAC. After exhaustive exercise and ischemia, uric acid is taken up by the muscles, most probably to be used as an intracellular antioxidant. In Hellsten et al. [36], they noticed this uptake of uric acid by the muscles is most probable in the 2nd and 10th min after ischemia. This is in agreement with our findings that TAC is slightly lower 10 min after strenuous exercise related to probable ischemia in muscles. Changes in TAC values were the same in both groups during the initial and final tests. Partial Eta^2^ = 0.074 at the final test indicates potentially significantly higher values in the experimental group after exercise. This can be explained as an effect of supplementation; less oxidative stress demands a lower antioxidant uptake by muscles.

It is suggested that damage to muscle cells caused by free radicals formed during exercise may initialize inflammation [37]. This is the reason why we observed the influence of supplementation on inflammation parameters. We have chosen IL-6 as a cytokine which is one of the sensitive indicators of inflammation in the body and CRP as one of the proteins of the acute phase used for estimation of the nonspecific inflammation process in everyday practice [38]. IL-6 is activated after binding to soluble receptors. This complex is believed to be a central regulator of immunological and inflammatory processes in humans [39,40]. Acute phase responses to stress in the liver are also induced by IL-6, which includes the synthesis of several unique hepatic proteins. C-reactive protein (CRP) is an example of an acute phase protein and is a sensitive marker of inflammation regardless of etiology. It has been believed that the synthesis of CRP is induced by both IL-6 and IL-1. However, recently some studies found that basal CRP level in healthy individuals is mostly regulated by IL-1 [41,42]. The release of IL-6 after exercise is caused mainly by the working skeletal muscle, although it can be secreted by different tissues [43]. In our study, IL-6 level was similar in both groups before exercise (exp.gr. 15.87 ± 3.23 pg/mL, cont.gr. 15.29 ± 3.35 pg/mL) and strenuous physical activity caused similar elevation of the IL-6 in both groups (exp.gr 17.77 ± 2.48 pg/mL, cont.gr. 17.49 ± 2.97 pg/mL). However, after 6 weeks of supplementation, there was a statistically significant decrease in IL-6 in the experimental group before (*p* = 0.035) and after (*p* = 0.050) the ergometer testing compared to the control group. A lower baseline level of IL-6 in the experimental group after supplementation indicates, similar to CK, a positive influence on the recovery process after intensive exercise related to training, decreasing inflammation reaction caused by oxidative stress. However, significantly lower IL-6 after ergometer testing appears to be due to a protective effect on acute muscle damage.

CRP level was a little bit, not significantly, higher in the experimental group at initial testing but no physical activity or supplementation had a significant influence on this biochemical parameter. A study similar to ours with Polish national rowers has proved the beneficial effect of GliSODin supplementation on inflammation levels using exactly CRP as a marker. In this study 2000-m maximal effort ergometer test did not influence the CRP level, but after 6 weeks of the basic supplementation level of CRP before ergometer testing was significantly lower in the experimental group. This finding is not coincident with ours, even though there was some decrease in CRP level in the final test in the experimental group but significance was absent [44]. Some studies have evidenced that there is no correlation between the level of IL-6 and CRP after strenuous exercise, where even IL-6 level was elevated after cycling to exhaustion; CRP was unchanged [45]. Studies that have investigated changes in levels of an inflammatory marker, including CRP, after intensive exercise in men conclude that the rise in CRP levels after intensive exercise may be time-lagged [46]. Some studies observed an increase in plasma CRP levels as late as 7 days after exercise in triathletes [47]. Finally, Miles et al. [48] reported a rise in CRP levels 4 h after a 32-km mountain trial race, and CRP concentrations further increased after 24-h restitution. After these findings, we can assume that in our case, it is possible that the CRP level rose later after physical activity, not in the first 10 min. To gain a better perspective of the anti-inflammation effect of the supplement, it would be very interesting to examine more inflammation parameters (IL-8, IL-10, TNF-α, cortisol) during 24 h after ergometer testing.

To estimate the rowing performance of athletes included in our study, we have been measuring maximal blood lactate concentration after the ergometer testing, maximally achieved power (workload), and rowing power at a lactate concentration of 4 mmol/L. Lucia et al. [49] concluded that the main physiologic advantage of professional athletes was their ability to maintain very high workloads without intolerably high lactate concentrations in their blood. In reality, this likely represents superiority in both oxygen delivery and lactate utilization process in their body. In our study, maximal lactate concentration, maximal power in ergometer testing, and estimated power at 4 mmol/L lactate concentration were used as parameters of performance and endurance (Table 6). There were no significant differences between groups at initial testing, same as at final testing in maximal lactate concentration and maximal achieved power (W max). These results are not very surprising because training during the period of 6 weeks of basic preparation was not specifically planned for every rower to enhance specific, i.e., competitive rowing abilities. It is likely that the basic, i.e., predominantly low and middle-intensity aerobic training, even provokes positive adaptation (Figure 1, ΔLa peak = 0.54 ± 1.55 vs. 1.14 ± 1.44%, ΔW max = 5.86 ± 20.92 vs. 5.72 ± 8.38% Experimental vs. Control group Initial vs. Final test, respectively), did not result in statistically significant metabolic adaptation in the subjects in terms of increasing the production of maximally achieved anaerobic acidosis (Table 6, La peak—Experimental group = 14.95 ± 1.52 vs. 15.39 ± 1.55 and Control group = 14.23 ± 1.61 vs. 15.37 ± 1.84 mmol/L Initial vs. Final test, respectively), or in terms of maximally achieved rowing power (Table 6, W max—Experimental group = 453.21 ± 82.06 vs. 459.07 ± 78.67 and Control group = 439.37 ± 83.84 vs. 445.04 ± 82.84 Watt Initial vs. Final test, respectively). However, when it comes to the level of rowing power at 4 mmol/L lactate concentration after supplementation, we can notice statistically significantly better results in the experimental group (Table 6, W at 4 mmol/L—Experimental group = 270.69 ± 47.41 vs. 281.30 ± 47.49 and Control group = 269.18 ± 50.23 vs. 266.53 ± 49.57 Watt Initial vs. Final test, respectively). Although in the control group, after 6 weeks of training, a slight decrease in rowing power at a metabolic load of 4 mmol/L was observed (which is a possible consequence of different individual adaptive abilities of the subjects in the group or different states of cumulative fatigue after 6-week training), however, the experimental group showed statistically significantly higher rowing power at the same load (Figure 1, 10.66% vs. −2.65%, experimental vs. Control group, respectively). In other words, it can be considered that supplementation with GliSODin could provide better exercise performance for professional athletes at an intensity of OBLA, i.e., 4 mmol/L of metabolic acidosis. These results, it seems, indicate that GliSODin could be used as good nutritional support under conditions of prolonged and strenuous physical activity, suppressing adverse effects of oxidative stress on muscle damage, inflammation, and sports performance.

## 5. Conclusions

According to these results, we can conclude that supplementation with antioxidant GliSODin 500 mg per day in a population of elite rowers during 6 weeks of mesocycle of basic preparing period had a significant influence on the parameters of muscle damage, inflammation, and sports performance. Despite the fact that GliSODin is an antioxidant supplement, during the 6 weeks of applied supplementations, we did not find any statistically significant changes in total antioxidant capacity, but we did notice lower initial values of CK and IL-6 and enhanced power at 4 mmol/L lactate concentration. The medium effect size of positive change of CRP, LDH, and TAC are also encouraging for future studies. The limitation of this study was the small number of participants and duration of the study. The longer period of supplementation would enable cumulative effects, which could become apparent in performance outcomes. Moreover, the study was conducted during the basic preparation period when rowers did not have very severe, specifically intensive, training sessions. It would be interesting for further investigation to consider supplementation during exhausting training sessions before and during the competition period when athletes are more likely exposed to oxidative stress. Moreover, more controlled conditions concerning nutrition and free time activities would exclude possible interactions with food and lack of rest periods. This could provide a better quality of obtained results of a study. These positive results in the supplemented group are the reason why it can be concluded that further studies are needed with a large number of participants during longer periods of time in more controlled conditions to confirm the beneficial activity of GliSODin in athletes, protecting them from adverse effects of oxidative stress.

## Figures and Tables

**Table 1:** GliSODin ingredients.

Usual Name	Latin Binomial	Plant Part	CAS #
Melon concentrate	*Cucumis melo* L.	Fruit pulp	90063-94-8
Gliadin	*Triticum vulgare*	Wheat grain	9007-90-3
Maltodextrin	*Triticum* spp.	Wheat grain	9050-36-6

**Table 2:** Anthropometric and training data.

Parameter	Experimental Group (*n* = 15)	Control Group (*n* = 13)	Differences
Age (years)	25.5 ± 5.4	21.8 ± 5.5	*p* = 0.081
Height (m)	1.86 ± 0.08	1.87 ± 0.07	*p* = 0.667
Body weight (kg)	86.4 ± 8.7	82.1 ± 10.9	*p* = 0.256
Years of training	8.5 ± 5.0	7.3 ± 5.7	*p* = 0.551
Training duration per day (h)	2.05 ± 0.36	2.10 ± 0.47	*p* = 0.778

**Table 3:** Changes in biochemical parameters during the initial test before supplementation.

	Initial Test			
Differences	T1 (before test)		T2 (after test)	
**Wilks’ Lambda Value**	0.855		0.925	
**F**	0.744		0.357	
***p***	0.599		0.872	
**Partial Eta^2^**	0.145		0.075	
	Experimental group (*n* = 15)	Control group (*n* = 13)	Experimental group (*n* = 15)	Control group (*n* = 13)
CK (U/L)	173 ± 125	209 ± 102	225 ± 152	266 ± 109
	*p* = 0.418, Part. Eta^2^ = 0.025		*p* = 0.426, Part. Eta^2^ = 0.025	
LDH (U/L)	148 ± 21	147 ± 16	177 ± 23	180 ± 17
	*p* = 0.891, Part. Eta^2^ = 0.001		*p* = 0.662, Part. Eta^2^ = 0.007	
TAC (mmol/L)	4.26 ± 0.43	4.11 ± 0.45	4.08 ± 0.57	4.07 ± 0.66
	*p* = 0.370, Part. Eta^2^ = 0.031		*p* = 0.974, Part. Eta^2^ ≤ 0.001	
IL-6 (pg/mL)	15.87 ± 3.23	15.29 ± 3.35	17.77 ± 2.48	17.49 ± 2.97
	*p* = 0.648, Part. Eta^2^ = 0.008		*p* = 0.788, Part. Eta^2^ = 0.003	
CRP (mg/L)	0.88 ± 0.64	0.62 ± 0.28	0.93 ± 0.66	0.68 ± 0.34
	*p* = 0.175, Part. Eta^2^ = 0.070		*p* = 0.221, Part. Eta^2^ = 0.057	

Partial Eta^2^ = 0.01 indicates a small effect, Partial Eta^2^ = 0.06 indicates a medium effect, Partial Eta^2^ = 0.14 indicates a large effect. *p* ˂ 0.05, statistical significance.

**Table 4:** Changes in biochemical parameters during ergometer test after 6 weeks of supplementation.

	Final Test			
Differences	T1 (before test)		T2 (after test)	
**Wilks’ Lambda Value**	0.515		0.661	
**F**	4.151		2.253	
***p***	0.008		0.085	
**Partial Eta^2^**	0.485		0.339	
	Experimental group (*n* = 15)	Control group (*n* = 13)	Experimental group (*n* = 15)	Control group (*n* = 13)
CK (U/L)	152 ± 63	215 ± 101	197 ± 82	249 ± 98
	*p* = 0.049, Part. Eta^2^ = 0.137		*p* = 0.139, Part. Eta^2^ = 0.082	
LDH (U/L)	145 ± 19	155 ± 19	175 ± 20	185 ± 22
	*p* = 0.201, Part. Eta^2^ = 0.062		*p* = 0.245, Part. Eta^2^ = 0.052	
TAC (mmol/L)	4.34 ± 0.57	4.17 ± 0.43	4.12 ± 0.55	3.81 ± 0.59
	*p* = 0.391, Part. Eta^2^ = 0.028		*p* = 0.162, Part. Eta^2^ = 0.074	
IL-6 (pg/mL)	10.59 ± 5.67	14.44 ± 2.83	13.33 ± 5.65	16.64 ± 1.85
	*p* = 0.035, Part. Eta^2^ = 0.159		*p* = 0.050, Part. Eta^2^ = 0.136	
CRP (mg/L)	0.68 ± 0.41	0.62 ± 0.38	0.68 ± 0.34	0.66 ± 0.41
	*p* = 0.677, Part. Eta^2^ = 0.007		*p* = 0.897, Part. Eta^2^ = 0.001	

Partial Eta^2^ = 0.01 indicates a small effect, Partial Eta^2^ = 0.06 indicates a medium effect, Partial Eta^2^ = 0.14 indicates a large effect. *p* ˂ 0.05, statistical significance.

**Table 5:** Changes in measured biochemical parameters due to specific maximal effort physical activity according to tests and experimental groups.

	ΔCK (%)	ΔLDH (%)	ΔTAC (%)	ΔIL-6 (%)	ΔCRP (%)
Initial test					
Exp. group	32.95 ± 13.94	20.90 ± 12.62	−1.0 ± 12.0	14.37 ± 16.45	4.91 ± 8.78
Control group	32.37 ± 15.96	23.86 ± 10.45	−3.80 ± 14.03	19.33 ± 31.15	6.27 ± 14.51
*p*-values	0.919	0.509	0.579	0.596	0.763
Final test					
Exp. group	30.28 ± 9.2	21.37 ± 9.36	−3.83 ± 14.64	47.51 ± 54.74	2.24 ± 11.1
Control group	20.2 ± 14.14	19.71 ± 5.84	−7.79 ± 15.02	18.06 ± 17.44	5.14 ± 8.72
*p*-values	0.032	0.586	0.486	0.075	0.454
Initial vs. Final test					
Exp. group	−2.67 ± 11.52	0.47 ± 18.40	−0.027 ± 18.16	33.14 ± 50.57	−2.67 ± 13.30
Control group	−12.17 ± 3.85	−4.13 ± 9.442	−6.79 ± 14.26	−1.27 ± 21.24	−1.12 ± 12.74
*p*-values	0.009	0.423	0.289	0.031	0.757

**Table 6:** Sport performance parameters changes during the ergometer test before and after supplementation.

	Initial Test		Final Test	
	Experimental group (*n* = 15)	Control group (*n* = 13)	Experimental group (*n* = 15)	Control group (*n* = 13)
La peak (mmol/L)	14.85 ± 1.52	14.23 ± 1.61	15.39 ± 1.55	15.37 ±1.84
	*p* = 0.299, Part. Eta^2^ = 0.041		*p* = 0.978, Part. Eta^2^ ˂ 0.001	
W max (Watt)	453.21 ± 82.06	439.37 ± 83.84	459.07 ± 78.67	445.04 ± 82.84
	*p* = 0.663, Part. Eta^2^ = 0.007		*p* = 0.650, Part. Eta^2^ = 0.008	
W at 4 mmol/L La (Watt)	270.69 ± 47.41	269.18 ± 50.23	281.30 ± 47.49	266.53 ± 49.57
	*p* = 0.936, Part. Eta^2^ ˂ 0.001		*p* = 0.429, Part. Eta^2^ = 0.024	

Partial Eta^2^ = 0.01 indicates a small effect, Partial Eta^2^ = 0.06 indicates a medium effect, Partial Eta^2^ = 0.14 indicates a large effect. *p* ˂ 0.05, statistical significance.

## Data Availability

Not applicable.
